# Evaluation of the quickmic system in the rapid diagnosis of Gram-negative bacilli bacteremia

**DOI:** 10.1128/spectrum.04011-23

**Published:** 2024-08-28

**Authors:** Celia García-Rivera, Andrea Ricart-Silvestre, Mónica Parra Grande, María Paz Ventero, Iryna Tyshkovska-Germak, Antonia Sánchez-Bautista, Esperanza Merino, Juan Carlos Rodríguez

**Affiliations:** 1 Department of Microbiology, Dr. Balmis University General Hospital, Alicante Institute for Health and Biomedical Research (ISABIAL), Alicante, Spain; 2 Infectious Diseases Unit, Dr. Balmis University General Hospital, Alicante Institute for Health and Biomedical Research (ISABIAL), Alicante, Spain; 3 Miguel Hernandez University, Alicante, Spain; London Health Sciences Centre, London, Ontario, Canada

**Keywords:** QuickMIC, rapid phenotypic system, antibiogram, Gram-negative bacilli, bacteremia

## Abstract

Rapid microbiological diagnosis of the antibiotic susceptibility of Gram-negative bacilli is a priority in clinical microbiology, especially in cases of bacteremia. The rapid advancement of antimicrobial resistance proposes a challenge for empirical antibiotic therapy and shows the need for fast antibiotic susceptibility diagnostics to guide treatments. The QuickMIC System (Gradientech AB, Uppsala, Sweden) is a recently developed rapid diagnostic tool for antibiotic susceptibility testing. Our study evaluates a rapid phenotypic system (QuickMIC) that provides information on the susceptibility of 12 antibiotics against *Escherichia coli*, *Klebsiella spp*., *Pseudomonas aeruginosa*, *Acinetobacter baumannii*, *Enterobacter cloacae*, *Proteus spp*., *Citrobacter spp.,* and *Serratia marcescens*. A total of 816 antibiotic/microorganism combinations were tested, resulting in eight discrepancies. The concordance between the antibiotics offered by QuickMIC and reference methods (MicroScan WalkAway plus system, Beckman Coulter; Etest (BioMerieux microdilution system (Bruker); Real-time PCR (GeneXpert, Cepheid); and immunochromatography (Biotech) was 99.02%. Time elapsed to obtain a valid minimal inhibitory concentration (MIC) was between 2 and 4 h. The QuickMIC system allows for the early adjustment of antibiotic treatment in these infections. Given the existing limitations of currently available rapid methods, its clinical utility is particularly relevant in the management of *P. aeruginosa* infections and AmpC-producing Enterobacterales. The use of rapid methods can help diversify antibiotic use and reduce carbapenem consumption.

**IMPORTANCE:**

The rapid diagnosis of antibiotic sensitivity in Gram-negative bacilli is of paramount importance in clinical microbiology, particularly in cases of bacteremia. The escalating challenge of antimicrobial resistance underscores the need for expeditious antibiotic susceptibility diagnostics to guide empirical antibiotic therapy effectively. In light of this, we present our study that evaluates the QuickMIC System, a recently developed rapid diagnostic antibiogram. QuickMIC System, offers a novel approach to phenotypic testing, providing information on the activity of 12 antibiotics against key pathogens, including *Escherichia coli*, *Klebsiella spp*., *Pseudomonas aeruginosa*, *Acinetobacter baumannii*, *Enterobacter cloacae*, *Proteus spp*., *Citrobacter spp*., and *Serratia marcescens*. Our investigation involved testing a total of 816 antibiotic/microorganism combinations. The study demonstrated an impressive 99.02% concordance between the QuickMIC System and the reference methods, with only eight discrepancies observed. The time to actionable minimum inhibitory concentration (MIC) ranged between 2 and 4 h, highlighting the system's efficiency in providing rapid results.

## INTRODUCTION

The rapid diagnosis of bacteremia caused by Gram-negative bacilli (GNR) is one of the priorities in clinical microbiology due to its significant clinical impact on patients and the frequency of these infections. An overall mortality rate of 11% has been reported, with inadequate treatment being a significant risk factor (1, 2). When the condition is associated with multidrug-resistant microorganisms, mortality rates can reach up to 50% (3).

Delayed administration of appropriate antibiotic treatment in bacteremia is associated with more unfavorable clinical outcomes, making it crucial to act quickly and efficiently in these situations (4). Early identification of the antibiotic susceptibility of microorganisms can significantly influence clinical outcomes, underscoring the need for rapid phenotypic assays to guide appropriate treatment (5–7). For this reason, various methods are being developed to provide information on the antibiotic susceptibility of microorganisms, analyzing both genotypic and phenotypic traits. In general, results show that the application of these techniques within multidisciplinary teams allows for the rapid identification of microorganisms and their antibiotic susceptibility, facilitating early treatment (8, 9). This can improve patient survival, reduce hospital stays, and decrease healthcare costs. Additionally, it reduces the use of broad-spectrum drugs, minimizing the increase in antibiotic resistance for the benefit of the patient (10).

*Escherichia coli* is the most frequent etiological agent of bacteremia (11), particularly when associated with extended-spectrum beta-lactamase-producing strains. Although less common, bacteremias associated with *Pseudomonas aeruginosa* are notable for their severity and the challenges they present for treatment due to increasing resistance. Even when following international guidelines, selecting the appropriate treatment can be difficult (12). Bacteremias associated with *Klebsiella pneumoniae* have also been shown to cause a mortality rate of 34% within 90 days (13).

A pilot study was previously conducted by the developers of the QuickMIC System (Gradientech AB, Uppsala, Sweden), a new system for the phenotypic study of the resistance patterns of the main Gram-negative bacilli associated with bacteremia, using samples from a geographic area with a low incidence of multidrug resistance (14). This study demonstrated that the QuickMIC system can provide antibiotic susceptibility testing (AST) data very rapidly for up to 12 antibiotics and at least 10 different species of Gram-negative bacteria, with an average time of approximately 3 h. Therefore, in this work, we continue the evaluation of this new system for phenotypic study within the routine clinical practice of a hospital with a high incidence of multidrug-resistant bacteria and a multidisciplinary management team.

## MATERIALS AND METHODS

### Design

An observational study was designed to evaluate the performance of the QuickMIC System (Gradientech AB, Uppsala, Sweden) in clinical practice at the General University Hospital, Dr Balmis (Alicante, Spain); Inclusion criteria: Monomicrobial bacteremia caused by Gram-negative bacilli, for which the new system was validated. Exclusion criteria: Bacteremia caused by other pathogens and poly-microbial/bacteremia.

### Samples

Eighty-six consecutive positive blood culture samples with Gram-negative bacilli bacteremia were collected from November 2022 to March 2023.

### Evaluation system

This is the first evaluation of the QuickMIC System in real clinical practice, following a pilot study conducted at Gradientech AB, Uppsala, Sweden (14). QuickMIC is an ultra-fast antibiogram system that directly utilizes positive blood culture samples. QuickMIC uses microfluidics, real-time microscopy, and light-scatter growth quantification, to provide bacterial susceptibility results within 2–4 h. The technology consists on the formation of a stable antibiotic gradient (in a three-dimensional agarose gel) covering a range of concentrations that increase linearly. Real-time imaging allows for monitorization of the bacterial growth rate and quantification of individual bacterial colonies, enabling a quick identification of the minimum inhibitory concentration (MIC).

The antibiotics analyzed in the current study using the QuickMIC GN cassette (cat. no: 43-001-10) included: amikacin, cefepime, ciprofloxacin, colistin, cefotaxime, ceftazidime/avibactam, ceftazidime, gentamicin, meropenem, piperacillin/tazobactam, tigecycline, and tobramycin.

The QuickMIC system is validated for the following microorganisms*: E. coli*, *Klebsiella spp*. *(K. pneumoniae*, *K. variicola*, *K. Oxytoca*, *and K. aerogenes), P. aeruginosa*, *Acinetobacter baumannii*, *Enterobacter cloacae*, *Proteus spp*. *(P. mirabilis and P. vulgaris)*, *Citrobacter spp*. *(C. koserii and C. freundii)*, and *Serratia marcescens*.

**Fig 1:**
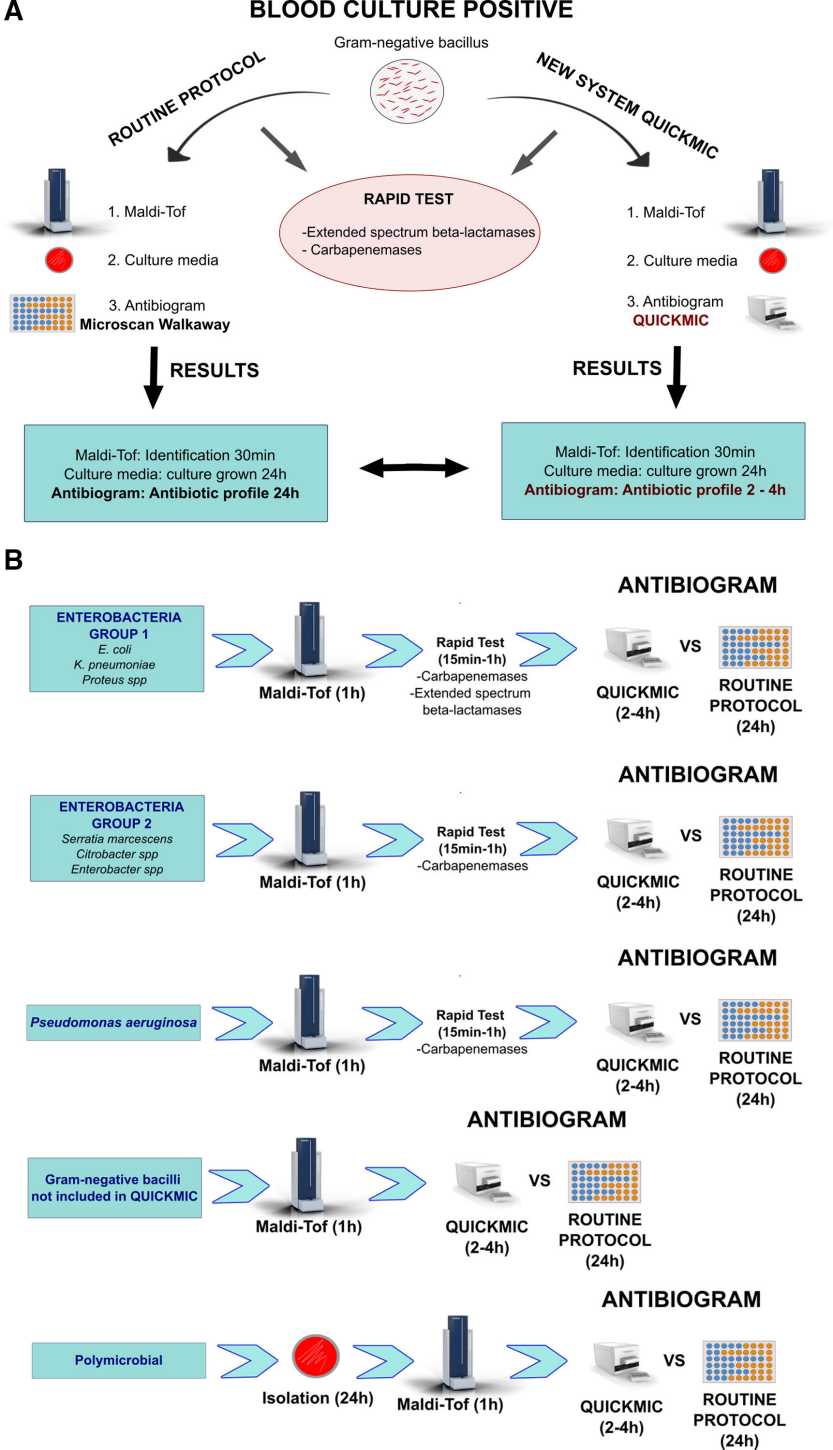
(**A**) Comparative scheme of the usual work protocol versus the new diagnostic method. (**B**) Comparative scheme of the time of each procedure according to the type of microorganism (both methods).

Working protocol: The system was integrated into our protocol for the microbiological diagnosis of Gram-negative bacilli bacteremia (Fig. 1A). The routine protocol consisted first on the identification of the microorganism using Maldi-Tof from a positive blood culture by performing a series of centrifugations to obtain the identification in approximately 30 min. Later, real-time PCR (GeneXpert, Cepheid) and immunochromatography (Biotech) systems were used for the rapid detection of the main resistance mechanisms (extended-spectrum beta-lactamases and carbapenemases). Finally, culture media were seeded and an antibiogram was performed using the MicroScan WalkAway plus system (Beckman Coulter). Borderline or anomalous susceptibility results were confirmed using the E-test (BioMerieux) or a microdilution system (Bruker), especially for colistin susceptibility. The antibiotic susceptibility profile was obtained 24 h after the blood culture tested positive.

The new method was incorporated into the routine antibiotic susceptibility study protocol for evaluation. The new system provided antibiogram results within 2–4 h, compared to 24 h for the MicroScan WalkAway plus system. Figure 1B illustrates protocol modifications based on the type of microorganism obtained in the blood culture and situations where the new system can be utilized.

Antibiotic resistance pattern: With the data obtained on the antibiotic susceptibility of the microorganisms included in the study, the antibiotic resistance pattern was calculated using the EUCAST 2023 criteria for the susceptible or resistant category.

### Data analysis

A categorical agreement between QuickMIC and Microscan is presented. Results are classified based on the type of discrepancy, categorized as very major, major, or minor discrepancy. A very major discrepancy was defined as the new method reporting susceptible when the antibiotic was resistant. Conversely, a major discrepancy was identified when the new method classified the antibiotic as resistant despite it being susceptible. Minor discrepancies were considered when susceptible or resistant categories were reported as increased exposure (I) in the test system, or vice versa.

## RESULTS

Out of 86 samples, 18 corresponded to bacteremias caused by Gram-negative bacilli not included in the panel and/or polymicrobial bacteremias. These samples, accounting for 20.9%, were excluded from the study. Among the remaining 68 samples, the distribution was as follows: *E. coli* (*n* = 41, 60.3%), *Klebsiella spp*. (*n* = 14, 20.5%), *Enterobacter spp*. (*n* = 5, 7.4%), *P. aeruginosa* (*n* = 4, 5.9%), *Citrobacter spp*. (*n* = 2, 2.9%), *Proteus spp*. (*n* = 1, 1.5%), and *Serratia marcescens* (*n* = 1, 1.5%). The detailed antibiotic resistance pattern found is presented in Table 1. A total of 816 antibiotic/microorganism combinations were tested (12 for each sample), revealing eight discrepancies. This yielded a concordance rate of 99.02% between antibiotics. Detailed results are provided in Table 2.

**TABLE 1:** Resistance rates of isolates against the antibiotics tested^*a*^

Isolates, *N* = 61	Resistance rate (%)											
	AK	CE	CPF	CL	CFT	CFA	CTZ	GEN	ME	*P*/T	TIG	TO
*Escherichia coli* (41)	0	12.2	26.8	2,4	12.2	0	9.8	4.9	0	0	0	7.3
*Klebsiella pneumoniae* (13)	0	7.7	7.7	7.7	15.4	0	15.4	7.1	0	15.4	-	7.7
*S. marcescens* (1)	100	0	100	100	0	0	0	0	0	0	-	0
*P. aeruginosa* (4)	0	75	25	0	-	0	50	-	25	50	-	25
*Proteus spp.* (1)	0	0	0	100	0	0	0	0	0	0	-	0

Isolates with inducible AmpC genes, *N* = 7	Resistance rate (%)											
	AK	CE	CPF	CL	CFT	CFA	CTZ	GEN	ME	P/T	TIG	TO
*Enterobacter cloacae complex* (5)	0	25	25	0	50	0	50	25	0	25	-	25
*Citrobacter spp.* (2)	0	0	0	0	0	0	0	0	0	0	0	0
*Klebsiella aerogenes* (1)	0	0	0	0	0	0	0	0	0	0	0	0

^*a*^ AK: Amikacin, CE: cefepime, CPF: ciprofloxacin, CL: colistin, CFT: cefotaxime, CFA: ceftazidime/avibactam, CTZ: ceftazidime, GEN: gentamicin, ME: meropenem, *P*/T: piperacillin/tazobactam, TIG: tigecycline, TO: tobramycin. The EUCAST 2023 cutoff points were used to classify the microorganisms in the susceptible or resistant category to the different antibiotics.

**TABLE 2:** Discrepancies between the usual method and the new QuickMIC system^*a*^

Discrepancy												
Isolates	AK	CE	CPF	CL	CFT	CFA	CTZ	GEN	ME	P/T	TIG	TO
*Escherichia coli*	VMD (1)	VMD (2)	-	-	-	-	-	-	-	-	-	VMD (1)
*Klebsiella pneumoniae*	-	-	-	-	M (1)	-	VMD (1)	-	-	VMD (1)	-	-
*S. marcescens*	-	-	-	-	-	-	-	-	-	M (1)	-	-

^*a*^ VMD = Very major discrepancy, the isolate is resistant to the usual method and susceptible to the new method. M: major discrepancy, the isolate is susceptible to the usual method and resistant to the new method. AK: Amikacin, CE: cefepime, CPF: ciprofloxacin, CL: colistin, CFT: cefotaxime, CFA: ceftazidime/avibactam, CTZ: ceftazidime, GEN: gentamicin, ME: meropenem, *P*/T: piperacillin/tazobactam, TIG: tigecycline, TO: tobramycin.

## DISCUSSION

The evaluated system excels in rapidly providing susceptibility data for multiple antibiotics against the primary pathogens associated with Gram-negative bacilli bacteremia. Compared to traditional systems, it demonstrates higher sensitivity and specificity, with a significantly shorter turnaround time (4–6 h vs 17–24 h). It is especially useful for the early detection of microorganisms included in the recently constituted “difficult-to-treat resistance” category, as they are not susceptible to all first-line antibiotics (carbapenems, β-lactam-β-lactamase inhibitor combinations, and fluoroquinolones). The phenotypic results of the antibiogram obtained by QuickMIC facilitate the rapid implementation of appropriate measures to control the spread of these strains in the hospital environment (15–17).

The new system is particularly relevant for analyzing antibiotic susceptibility in Gram-negative bacilli against first-line medications, where rapid protein or gene-based tests are lacking. Thus, the rapid detection of resistance mechanisms in *P. aeruginosa* is notably limited by these methods, both against piperacillin/tazobactam, ceftazidime, or cefepime and against carbapenems or the new cephalosporins (ceftazidime/avibactam or ceftolozane/tazobactam). There is no rapid method available to detect resistance to any of the drugs mentioned, except in the case of strains producing carbapenemase. Therefore, the newly evaluated system provides valuable clinical information that is not typically provided by systems commonly distributed to clinical microbiology laboratories. This fact is very relevant; as rapid microbiological diagnosis has been seen to improve the management of patients with carbapenem-resistant *P. aeruginosa* (18–20).

Weaknesses include the inability to analyze polymicrobial infections or infections caused by microorganisms not validated for the system, typical of genotypic systems. Overall, it has been reported that 49.3% (*N* = 102) of polymicrobial cultures were incompletely identified by the FilmArray result (21). As market trends shift toward rapid microbiological diagnostics, evaluating these systems becomes imperative, considering local resistance epidemiology, stewardship team presence, and laboratory capabilities. These systems are especially beneficial in settings with high multidrug-resistant Gram-negative bacilli prevalence or serious infections in vulnerable patients (22–27).

In contrast, phenotypic studies of multiple antibiotics offer more comprehensive information compared to systems based on genotypic detection. This is due to the multitude of possible genetic variants, which increases the challenge of detecting less prevalent ones. For example, bacteria may produce extended-spectrum beta-lactamases other than CTX-M, or exhibit mechanisms of resistance to carbapenems unrelated to the production of the most prevalent carbapenemases. Additionally, some bacteria may be associated with hyperproduction of AmpC, affecting the analysis of beta-lactam drugs (28, 29). It is very difficult to detect the genetic mechanisms of resistance to other families of drugs, so phenotypic studies are much more important in this case (30, 31). In addition to the correct treatment of multidrug-resistant bacteria, information on antibiotics from multiple families allows to choose the antibiotic with the narrowest spectrum within a few hours (32). In fact, EUCAST has proposed a rapid phenotypic system for the analysis of positive blood cultures using antibiotic discs, but it is validated for a limited number of microorganisms and antibiotics; the Vitek system (Biomerieux) is also being evaluated, to shorten the response time in case of positive blood cultures, with good results (12, 33, 34).

It is imperative to emphasize the necessity for rapid and precise management of *P. aeruginosa* infections, given the limitations of current rapid diagnostic methods and the questioning of traditional treatment protocols for highly vulnerable patients, owing to the prevalence of multi-resistant strains (12). Furthermore, the introduction of the new system holds promise in reducing carbapenem usage by providing information on the activity of multiple antibiotics. This enables the selection of treatment tailored to the clinical situation, with a narrower spectrum and reduced ecological and microbiome impact (35).

In relation to the discrepancies found between the two systems, they are very few despite the use of different technologies, which demonstrates their high clinical concordance; it is known that the phenotypic analysis of antibiotic activity is greatly influenced by the genetic characteristics of microorganisms, variations in the inoculum preparation, and in the incubation time (36).

The system’s ability to rapidly provide reliable information on 12 antibiotics will curb broad-spectrum antibiotic use, controlling antibiotic resistance while facilitating personalized patient treatment. However, this must be included in a protocol for the diagnosis of Gram-negative bacilli bacteremia, so that, following the criteria established by the diagnostic stewardship, the use of this technique is performed at the right time and on the right patient (37, 38, 39) .

### Conclusions

The system evaluated provides rapid information on the antibiotic activity of 12 antibiotics in the main Gram-negative bacilli associated with bacteremia, and can therefore, provide very useful information for the early adjustment of antibiotic treatment, for these processes. It is particularly useful in the management of *P. aeruginosa* infections, because in these processes, the available rapid methods present significant limitations.
